# Serial plasma proenkephalin A 119–159 and dipeptidyl peptidase 3 after cardiac arrest: association with long-term neurological outcome

**DOI:** 10.1016/j.resplu.2026.101496

**Published:** 2026-09-17

**Authors:** Bergþóra Þorgeirsdóttir, Anna Lybeck, Hans Friberg, Attila Frigyesi

**Affiliations:** a Department of Clinical Sciences, Anaesthesiology and Intensive Care, Lund University, SE-22185 Lund, Sweden; b Skåne University Hospital, Department of Intensive and Perioperative Care, SE-20502 Malmö, Sweden; c Skåne University Hospital, Department of Intensive and Perioperative Care, SE-22185 Lund, Sweden

**Keywords:** Cardiac arrest, Proenkephalin, Dipeptidyl peptidase 3, Outcome, Neuroprognostication, Biomarkers

## Abstract

To evaluate whether serial plasma proenkephalin A 119–159 (penKid) and circulating dipeptidyl peptidase 3 (cDPP3) after cardiac arrest are associated with long-term neurological outcome and mortality. Methods: We performed a retrospective multicentre cohort study of adults admitted to intensive care after cardiac arrest in southern Sweden. Biomarkers were measured on admission and at 12 and 48 h. The primary outcome was poor neurological outcome, defined as Cerebral Performance Category 3–5 at 2–6 months. The secondary outcome was 180-day mortality. Models were adjusted for established *peri*-arrest prognostic variables. Odds ratios and hazard ratios are reported per 1-SD increase in log_10_transformed biomarker concentration. Results: Among 417 patients, 271 (65%) had poor neurological outcome and 266 (64%) died within 180 days. PenKid was independently associated with poor neurological outcome at admission (adjusted odds ratio 1.62, 95% confidence interval 1.15–2.28) and 12 h (1.50, 1.08–2.09), and with mortality at admission (adjusted hazard ratio 1.36, 1.20–1.54) and 12 h (1.33, 1.17–1.51). cDPP3 was independently associated with mortality at 12 h (1.26, 1.11–1.43), but not with poor neurological outcome after adjustment. Neither biomarker significantly improved discrimination beyond the clinical reference model. Conclusions: Early penKid was associated with poor neurological outcome and mortality after cardiac arrest, whereas cDPP3 was associated with mortality at 12 h. These biomarkers added limited discriminatory value beyond established clinical predictors.

## Highlights

• Serial penKid and cDPP3 were evaluated after cardiac arrest.
• Early penKid was associated with poor neurological outcome and death.
• cDPP3 at 12 h was independently associated with mortality.
• Neither biomarker improved AUC beyond clinical predictors.

## Introduction

Multimodal neurological prognostication using clinical examination, neurophysiological tests, biomarkers, and brain imaging is recommended for comatose patients no earlier than 72 h after cardiac arrest.^1^ Although neuron-specific enolase (NSE) is currently the only biomarker recommended in international guidelines, it lacks brain specificity, can be falsely elevated by hemolysis, and due to delayed kinetics it is not useful for early prognostication.^2^, ^3^ These limitations warrant further investigation for alternative or additional biomarkers.

Proenkephalin A119-159 (penKid) is a stable, non-functional prohormone fragment derived from the endogenous enkephalin precursor.^4^ The opioid enkephalin system is present both centrally and peripherally, including the adrenal medulla, heart, kidney and in the central nervous system, with concentrations about 100-fold higher in the cerebrospinal fluid (CSF) than in the serum.^5^, ^6^ As a small, unbound molecule it is freely filtered through the kidney glomerulus and closely correlates with glomerular filtration rate.^7^, ^8^ Unlike the mature enkephalins it shows stability in the plasma for at least 48 h, which is favourable for reliable quantification in biomarker studies.^6^ Elevated plasma levels have been associated with renal failure,^9^, ^10^ in acute and chronic heart failure,^11^, ^12^, ^13^ and poor neurological outcome after stroke,^14^ intracerebral hemorrhage,^15^ and cardiac arrest.^16^ Because it reflects both central and systemic injury, penKid may capture the complex pathophysiological burden after cardiac arrest.

Circulating dipeptidyl peptidase-3 (cDPP3) is a cytosolic zinc-dependent metallopeptidase released to the bloodstream following cellular injury.^17^ It is involved in the degradation of vasoactive peptides including angiotensin II, a key mediator in the renin-angiotensin-aldosterone system (RAAS), which may contribute to vasodilation and thus reduced blood pressure. Elevated plasma levels have been shown to be associated with hemodynamic instability and poor outcomes in sepsis,^18^ critically ill patients^19^ and acute heart failure.^20^ These findings suggest that it may both contribute to and indicate the extent of ischemia–reperfusion injury. Its rapid release after cellular injury and association with multiorgan failure^21^ make cDPP3 an interesting early biomarker for prognostication following cardiac arrest.

In a previous study, our group demonstrated that penKid and cDPP3 were associated with poor neurological outcome at hospital admission following cardiac arrest. Notably, penKid was independently associated with poor prognosis even after adjusting for key clinical variables.^16^ In the present study, we sought to further evaluate the prognostic value of serial penKid and cDPP3 measurements in a larger cohort with sampling at admission and at 12 and 48 h after cardiac arrest. Specifically, we examined their associations with long-term neurological outcome and 180-day mortality and explored the potential influence of WLST on these associations.

## Methods

### Study design and setting

In this retrospective, multicenter study, patients were admitted after cardiac arrest to four intensive care units (ICU) in southern Sweden from 2014 to 2018. The size of the study was not predetermined but defined by the number of eligible ICU admissions and the availability of valid plasma samples. Blood samples were consecutively stored in the SWECRIT biobank, a biobank established to support research in the critically ill.^22^, ^23^

A detailed description of the study cohort has been previously published,^24^ but differed slightly due to stricter inclusion criteria and sample availability. The present cohort partially overlaps with that of our earlier admission-only study^16^: the previous study evaluated a single admission measurement in patients admitted during 2016, whereas the present study includes patients admitted between 2014 and 2018 and required valid serial samples at admission, 12 and 48 h. The present work therefore addresses the distinct question of the temporal evolution and prognostic value of serial biomarker measurements.

### Participants

All adult patients (≥18 years) admitted to the ICU following cardiac arrest were eligible for inclusion. Patients received post-resuscitation care in accordance with international guidelines, including multimodal neuroprognostication performed no earlier than 72 h after cardiac arrest.^1^ Decisions regarding withdrawal of life-sustaining therapy (WLST) were made by the treating clinical teams based on multimodal assessment and were not protocolized for the study. PenKid and cDPP3 measurements were performed retrospectively and were therefore unavailable to treating clinicians. Blood samples were collected at predefined time points: ICU admission, 12 and 48 h after the cardiac arrest. Samples drawn more than 6 h outside the time points were considered invalid. Patients with only admission samples, or with follow-up samples collected outside these predefined time points, were excluded. Of the 417 included patients, 363 (87.1%) had a valid admission sample, 386 (92.6%) a valid 12-h sample and 263 (63.1%) a valid 48-h sample; all included patients had at least one valid 12-h or 48-h sample.

### Data sources and outcome

Patients were identified through the national ICU registry using international classification of diseases (ICD) code I46. Clinical data were collected from medical records, the International Cardiac Arrest Registry (INTCAR), the Swedish population register and the patient administrative system for intensive care units (PASIVA). The primary outcome was neurological status at two to six months following cardiac arrest, assessed by a healthcare professional. Cerebral Performance Category (CPC) scale^25^ was used, where a CPC score of 1–2 was categorized as good outcome and 3–5 as poor outcome. The secondary outcome was 180-day all-cause mortality after cardiac arrest.

### penKid and cDPP3 measurement

Blood was collected into ethylenediaminetetraacetic acid (EDTA) vacutainers at the predetermined time points, centrifuged, aliquoted, frozen and stored in the SWECRIT biobank at IDEON, Region Skåne, Sweden (registration number BD-47). The frozen samples were shipped to SphingoTec GmbH (Hennigsdorf, Germany), where penKid and cDPP3 were quantified using validated blinded chemiluminescence immunoassays.^26^, ^27^ Samples were collected, processed, aliquoted and stored at −80°C according to standardized biobank procedures and underwent identical handling regardless of admission date, minimizing the risk of differential pre-analytical bias related to storage time. Both analytes have demonstrated good analytical stability under frozen storage and repeated freeze–thaw cycles in the corresponding assay validation studies^26^, ^27^; although stability data spanning the full biobank storage period are not available, the standardized and uniform handling of all samples makes systematic, storage-related bias unlikely.

### Statistics

Baseline characteristics, cardiac arrest variables, biomarker concentrations, medical history, and outcome measures were summarized for the overall cohort and stratified by neurological outcome (good CPC 1–2 vs poor CPC 3–5) and by arrest location (OHCA vs IHCA). Continuous variables were presented as median with interquartile range (IQR) and group comparison was made using the Mann-Whitney *U* test. Categorical variables were summarized as counts and percentages and compared between groups using Fisher’s exact test. Missing data were handled using complete-case analysis; variables with missing values were retained in descriptive tables but excluded from hypothesis testing.

Associations between biomarker concentrations and poor neurological outcome (defined as CPC 3–5) were assessed using univariate and multivariate logistic regression. The reference model comprised established early prognostic covariates. Variables incorporated were age, sex, time to return of spontaneous circulation (ROSC), admission motor Glasgow Coma Scale (GCS), shockable rhythm, admission pupillary reflex, out-of-hospital cardiac arrest (OHCA) and bystander cardiopulmonary resuscitation (CPR). Predictive power of the biomarkers was assessed using the area under the receiver operating characteristic curve (AUC). The DeLong method was used to compare AUCs for paired data. Multivariable analyses were performed using complete-case data; therefore, the number of patients included in individual models varied according to biomarker availability at the respective sampling time point and completeness of covariate data. The number of patients and outcome events included in each multivariable model is reported in Table 2, Table 3. The primary analyses focused on predefined associations between serial penKid concentrations and poor neurological outcome and 180-day mortality. Analyses of cDPP3, subgroup analyses, cause-specific mortality analyses, and sensitivity analyses were considered exploratory. Because the number of patients and outcome events within individual centres was small, site-stratified prognostic models were not fitted, as such models would be statistically unstable and prone to over-fitting; potential inter-centre variation in WLST practice is instead addressed as a limitation. Benjamini–Hochberg adjustment was applied to multiple group comparisons in descriptive biomarker analyses, but no formal multiplicity adjustment was applied to regression analyses.

**Table 1:** Characteristics of the study population by neurological outcome.

	**Overall**	**Good CPC**	**Poor CPC**	***p*-value**
Number of patients, *n* (%)	417 (100)	146 (35.0)	271 (65.0)	

**General characteristics**				
Female sex, *n* (%)	115 (27.6)	34 (23.3)	81 (29.9)	0.17
Age, years, median (IQR)	69 (59–76)	65 (56–74)	70 (61–77)	*<*0.01
OHCA, *n* (%)	318 (76.3)	102 (69.9)	216 (79.7)	0.03

**Cardiac arrest variables**				
Time to ROSC, min, median (IQR)	20 (12–36)	15 (10–24)	25 (15–40)	*<*0.001
Witnessed cardiac arrest, *n* (%)	330 (79.1)	125 (85.6)	205 (75.6)	0.02
Bystander CPR, *n* (%)	335 (80.3)	130 (89.0)	205 (75.6)	*<*0.01
Adrenaline, *n* (%)	336 (80.6)	79 (54.1)	257 (94.8)	*<*0.001
Shockable rhythm, *n* (%)	194 (46.7)	102 (70.3)	92 (34.1)	*<*0.001
Admission motor GCS, median (IQR)	1 (1–3)	3 (1–5)	1 (1–1)	*<*0.001
Admission pupillary reflex absent, *n* (%)	147 (36.6)	20 (14.4)	127 (48.3)	*<*0.001

Biomarkers on admission penKid (pmol/L), median (IQR)	123 (81–179)	86 (62–127)	139 (100–193)	*<*0.001
cDPP3 (ng/mL), median (IQR)	55 (36–83)	44 (32–65)	63 (40–92)	*<*0.001
Lactate (mmol/L), median (IQR)	8.1 (4.9–10.9)	5.9 (3.4–9.0)	9.1 (6.0–11.7)	*<*0.001
pH, median (IQR)	7.18 (7.01–7.27) 7.24 (7.13–7.31) 7.12 (6.95–7.25)			*<*0.001

Biomarkers at 12 h penKid (pmol/L), median (IQR)	102 (71–168)	83 (59–117)	114 (78–190)	*<*0.001
cDPP3 (ng/mL), median (IQR)	42 (27–75)	31 (24–50)	48 (30–84)	*<*0.001

Biomarkers at 48 h penKid (pmol/L), median (IQR)	62 (42–105)	57 (42–72)	74 (42–117)	0.04
cDPP3 (ng/mL), median (IQR)	23 (16–34)	23 (17–30)	23 (16–37)	0.55

**Previous medical history**				
Myocardial infarction, *n* (%)	69 (16.5)	29 (19.9)	40 (14.8)	0.21
Congestive heart failure, *n* (%)	76 (18.2)	28 (19.2)	48 (17.7)	0.79
Hypertension, *n* (%)	168 (40.3)	54 (37.0)	114 (42.1)	0.35
Liver disease, *n* (%)	10 (2.4)	5 (3.4)	5 (1.8)	0.33
Diabetes, insulin dependent, *n* (%)	50 (12.0)	14 (9.6)	36 (13.3)	0.34
Diabetes, non-insulin, *n* (%)	53 (12.7)	17 (11.6)	36 (13.3)	0.76
Cerebrovascular disease, *n* (%)	40 (9.6)	12 (8.2)	28 (10.3)	0.60
Solid tumour, *n* (%)	46 (11.0)	17 (11.6)	29 (10.7)	0.75
Renal disease, *n* (%)	41 (9.8)	10 (6.8)	31 (11.4)	0.17
Dementia/cognitive impairment, *n* (%)	19 (4.6)	3 (2.1)	16 (5.9)	0.09

**Patient outcome**				
ICU LOS, days, median (IQR)	3.2 (1.8–5.0)	2.1 (1.4–4.0)	3.7 (2.2–5.3)	<0*.*001
Hospital LOS, days, median (IQR)	7 (3–13)	14 (10–25)	4 (2–7)	<0*.*001
WLST, *n* (%)	209 (50.1)	0 (0)	209 (77.1)	*<*0.001
180-day mortality, *n* (%)	266 (63.8)	2 (1.37)	264 (97.4)	<0*.*001

*Values are presented as median (IQR) for continuous variables and n (%) for categorical variables. Good outcome defined as CPC 1–2, poor outcome as CPC 3–5. Continuous variables compared using Mann-Whitney U test and categorical variables using Fisher’s exact test. ROSC, return of spontaneous circulation; CPR, cardiopulmonary resuscitation; GCS, Glasgow Coma Scale; ICU, intensive care unit; LOS, length of stay; WLST, withdrawal of life-sustaining therapies; CPC, Cerebral Performance Category*.

**Table 2:** Discriminatory performance of penKid, cDPP3, and the clinical reference model for prediction of poor neurological outcome. ROC curve analyses were performed at admission, 12 h, and 48 h after cardiac arrest. The clinical reference model included age, sex, time to ROSC, motor GCS, shockable rhythm, pupillary reflex, bystander CPR, and arrest location (IHCA versus OHCA). Biomarker-augmented models were compared with the reference model using DeLong’s test. Values are presented as AUC with 95% confidence intervals (CI).

**Time point**	**Model**	***N***	**Events**	**AUC (95% CI)**	***p* vs reference**
Admission	Reference	345	227	0.89 (0.86–0.93)	–
	penKid only	345	227	0.73 (0.67–0.79)	–
	cDPP3 only	345	227	0.64 (0.57–0.70)	–
	Reference + penKid	345	227	0.90 (0.87–0.93)	0.15
	Reference + cDPP3	345	227	0.89 (0.86–0.93)	0.86

12 h	Reference	363	240	0.90 (0.86–0.93)	–
	penKid only	363	240	0.66 (0.60–0.72)	–
	cDPP3 only	363	240	0.66 (0.60–0.72)	–
	Reference + penKid	363	240	0.90 (0.87–0.93)	0.33
	Reference + cDPP3	363	240	0.90 (0.86–0.93)	0.36

48 h	Reference	248	170	0.89 (0.84–0.93)	–
	penKid only	248	170	0.59 (0.52–0.66)	–
	cDPP3 only	248	170	0.52 (0.44–0.59)	–
	Reference + penKid	248	170	0.89 (0.85–0.93)	1.00
	Reference + cDPP3	248	170	0.89 (0.85–0.93)	0.42

*AUC, area under the receiver operating characteristic curve; CPR, cardiopulmonary resuscitation; CI, confidence interval; cDPP3, circulating dipeptidyl peptidase 3; GCS, Glasgow Coma Scale; IHCA, in-hospital cardiac arrest; OHCA, out-of-hospital cardiac arrest; penKid, plasma proenkephalin A 119–159; ROC, receiver operating characteristic; ROSC, return of spontaneous circulation*.

**Table 3:** Cox proportional hazards regression analyses for 180-day all-cause mortality. Associations between plasma biomarker concentrations and 180-day mortality were evaluated using univariable and multivariable Cox proportional hazards models at admission, 12 h, and 48 h after cardiac arrest. For the primary analyses, follow-up time was calculated from cardiac arrest until death or censoring at 180 days. No left truncation was applied. Multivariable models were adjusted for age, sex, time to ROSC, motor GCS, shockable rhythm, pupillary reflex, arrest location (IHCA versus OHCA), and bystander CPR. Biomarkers were log_10_-transformed; HRs are reported per 1 SD increase in log_10_-transformed biomarker concentration.

**Time point**	**Biomarker**	***N***	**Events**	**HR**	**95% CI**	***p*-value**
**Univariable Cox models (180-day all-cause mortality)**						
0 h	penKid	370	240	1.55	1.39–1.71	<0.001
0 h	cDPP3	370	240	1.44	1.28–1.63	<0.001
12 h	penKid	386	249	1.47	1.31–1.64	<0.001
12 h	cDPP3	386	249	1.49	1.34–1.65	<0.001
48 h	penKid	263	176	1.22	1.05–1.41	0.01
48 h	cDPP3	263	176	1.14	0.99–1.31	0.07

**Multivariable Cox models (adjusted clinical reference model)**						
0 h	penKid	345	225	1.36	1.20–1.54	<0.001
0 h	cDPP3	345	225	1.14	0.99–1.31	0.08
12 h	penKid	363	239	1.33	1.17–1.51	<0.001
12 h	cDPP3	363	239	1.26	1.11–1.43	<0.001
48 h	penKid	248	168	1.11	0.94–1.31	0.21
48 h	cDPP3	248	168	0.99	0.85–1.15	0.86

*CI, confidence interval; CPR, cardiopulmonary resuscitation; cDPP3, circulating dipeptidyl peptidase 3; GCS, Glasgow Coma Scale; HR, hazard ratio; IHCA, in-hospital cardiac arrest; OHCA, out-of-hospital cardiac arrest; penKid, plasma proenkephalin A 119–159; ROSC, return of spontaneous circulation*.

Internal validation of the logistic regression models was performed using bootstrap resampling with 500 repetitions. Optimism-corrected AUCs were calculated by subtracting the mean bootstrap optimism from the apparent AUC. Incremental biomarker value beyond the clinical reference model was assessed using likelihood-ratio tests comparing nested models. Model calibration was evaluated using calibration plots.

Serum creatinine was excluded from the primary penKid reference model because admission creatinine data were incomplete, which would have reduced the analysable cohort, and because penKid is closely related to renal function, making adjustment a potential over-adjustment. Sensitivity analyses including creatinine were performed to confirm the robustness of the results.

Biomarker trajectories over time were analysed using linear mixed-effects models with biomarker concentration as the dependent variable and neurological outcome, time after cardiac arrest, and their interaction as independent variables.

A prespecified sensitivity analysis was conducted excluding patients in whom withdrawal of life-sustaining therapies (WLST) occurred, to reduce potential bias from self-fulfilling prognostication.

For 180-day all-cause mortality, survival probabilities were estimated using the Kaplan-Meier method and compared with the log-rank test. Associations between biomarker concentrations and mortality were assessed using Cox proportional-hazards regression, adjusted for the same reference model as in the logistic regression. For the primary analyses, follow-up was calculated from the time of cardiac arrest until death or 180 days.

As a sensitivity analysis, landmark Cox regression models were fitted at 12 and 48 h after cardiac arrest. Only patients alive at the respective landmark and with a valid biomarker measurement were included. Follow-up started at the landmark and continued until death or 180 days. The incremental prognostic value of penKid and cDPP3 beyond the reference model was evaluated using likelihood-ratio tests.

Exploratory competing-risk analysis was performed using cause-specific Cox proportional hazards models, classifying deaths as neurological or non-neurological. Classification of cause of death was performed before biomarker analysis and was therefore blinded to penKid and cDPP3 concentrations.

To assess missingness at 48 h, patients with and without a valid 48-h sample were compared with respect to baseline characteristics, markers of cardiac-arrest severity, neurological outcome, early mortality, and ICU length of stay. Continuous and ordinal variables were compared using the Wilcoxon rank-sum test and categorical variables using Fisher’s exact test.

Since plasma concentrations of penKid and cDPP3 were right-skewed, biomarker values were log_10_-transformed prior to statistical analysis. All biomarker ORs and HRs are reported per 1-SD increase in log_10_-transformed biomarker concentration. *P*-values < 0.05 were considered statistically significant. All statistical analyses were performed in R (R Foundation for Statistical Computing, Vienna, Austria).^28^

## Results

### Descriptive data

During the study period, 797 patients were admitted to the four ICUs with cardiac arrest among whom 417 met the inclusion criteria (Fig. S1). Of these, 318 (76.3%) had an OHCA and 99 (23.7%) an IHCA. The median age was 69 (IQR 59–76) years and 27.6% were female. At two to six month follow-up, 90 patients (21.6%) had CPC 1, 56 (13.4%) had CPC 2, 5 (1.2%) had CPC 3, 0 had CPC 4 and 266 (63.8%) had CPC 5. Life-sustaining therapies were withdrawn in 209 patients (50.1%), accounting for 77.1% of all patients with a poor neurological outcome.

Baseline characteristics stratified by arrest location (IHCA vs OHCA) are presented in Table S1, and stratified by neurological outcome (good vs poor CPC) in Table 1. As expected, OHCA patients had a longer time to ROSC (median 25 vs 10 min, *p <* 0*.*001), a higher proportion of shockable rhythm and more often absent pupillary reflexes at admission.

Patients with and without a valid 48-h sample were generally similar with respect to baseline characteristics and markers of cardiac-arrest severity (Supplementary Table S11). However, early clinical course differed. Among patients without a valid 48-h sample, 54/154 (35.1%) died within 48 h, compared with 12/263 (4.6%) among those with a valid sample (*p* < 0.001). Conversely, 53/154 (34.4%) patients without a valid sample had an ICU stay of ≤2 days without death within 48 h, compared with 28/263 (10.6%) of those with a valid sample (*p* < 0.001). Median ICU length of stay was also shorter among patients without a valid 48-h sample (1.6 [IQR 1.0–2.8] vs. 4.0 [2.8–5.7] days; *p* < 0.001). Poor neurological outcome and 180-day mortality did not differ significantly between groups.

### Plasma levels of penKid and its performance for prediction of outcomes

Levels of penKid in plasma were highest on admission and 12 h, with lower concentrations at 48 h as shown in Fig. 1. They were significantly increased in patients with poor neurological outcome at all time points as seen in Table 1. In subgroup analyses, penKid was significantly elevated in OHCA patients with poor neurological outcome at all time points, whereas no statistically significant differences were observed in the smaller IHCA subgroup (*n* = 99), in which statistical power was limited (see Fig. 1). Longitudinal biomarker trajectories according to arrest location and neurological outcome are shown in Figure S2. In mixed-effects models a significant time × outcome interaction was observed for penKid among OHCA patients (*p* = 0*.*02), but not among IHCA patients (*p* = 0*.*73). In univariate logistic regression analyses, increasing plasma penKid concentrations were associated with poor neurological outcome at all time points, with an OR of 2.49 (95% CI 1.87–3.32, *p <* 0*.*001) on admission, OR 1.78 (95% CI 1.38–2.28, *p <* 0*.*001) at 12 h, and OR 1.38 (95% CI 1.03–1.84, *p* = 0*.*03) at 48 h. After adjustment for age, sex, time to ROSC, motor GCS, shockable rhythm, pupillary reflex, OHCA and bystander CPR, increasing penKid concentrations remained independently associated with poor neurological outcome on admission, with an adjusted OR of 1.62 (95% CI 1.15–2.28, *p* = 0*.*01), and at 12 h, with an adjusted OR of 1.50 (95% CI 1.08–2.09, *p* = 0*.*02), but not at 48 h, as demonstrated in Table S2. Using only the clinical covariates, the reference model demonstrated high discriminatory performance with an AUC of approximately 0.89–0.90 across all time points. Addition of penKid to the model did not significantly improve predictive performance at any time point as shown in Fig. 3 and Table 2.

**Fig. 1:**
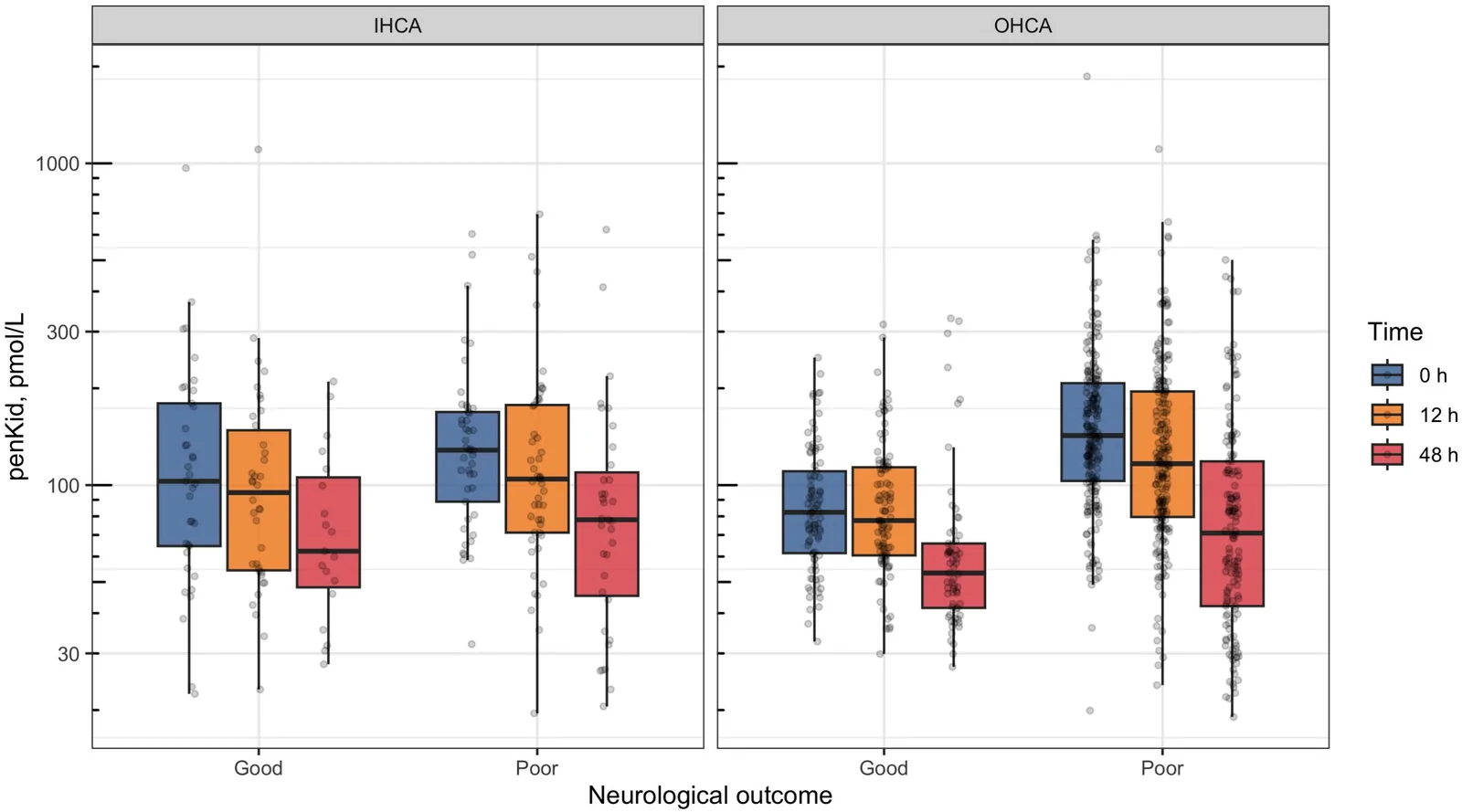
**Plasma penKid by neurological outcome, stratified by arrest location. Boxplots show plasma penKid concentrations at ICU admission (0 h), 12 h and 48 h for patients with good (CPC 1–2) and poor (CPC 3–5) outcome, stratified by IHCA and OHCA. Benjamini–Hochberg-adjusted *p*-values from Welch *t*-tests on log_10_ values: IHCA 0.3/0.42/0.62, OHCA <0.001/*<*0.001/0*.*06 at 0, 12 and 48 h, respectively**. *penKid, proenkephalin A 119–159; CPC, Cerebral Performance Category; IHCA, in-hospital cardiac arrest; OHCA, out-of-hospital cardiac arrest*.

### Plasma levels of cDPP3 and its performance for prediction of outcomes

Plasma levels of cDPP3 were highest on admission and decreased over time, as demonstrated in Fig. 2. cDPP3 concentrations were significantly elevated in patients with poor neurological outcome on admission and at 12 h, but not at 48 h (see Table 1). In subgroup analyses, significantly higher cDPP3 concentrations were observed in OHCA patients with poor neurological outcome on admission and at 12 h, whereas no statistically significant differences were observed in the IHCA group (see Fig. 2). Mixed-effects models showed a significant time × outcome interaction for cDPP3 in OHCA (*p* = 0*.*03) but not in IHCA patients (*p* = 0*.*94), see Figure S2. Univariate associations with poor outcome and multivariate models adjusting for clinical covariates are presented in Table S2. In univariate logistic regression analyses, increasing plasma cDPP3 concentrations were associated with poor neurological outcome on admission, with an OR of 1.68 (95% CI 1.29–2.19, *p <* 0*.*001), and at 12 h, with an OR of 2.00 (95% CI 1.49–2.68, *p <* 0*.*001), but not at 48 h. After adjustment for the predefined clinical covariates, cDPP3 was not independently associated with poor neurological outcome at any time point nor did its addition to the reference model significantly improve its prognostic performance, see Table 2.

**Fig. 2:**
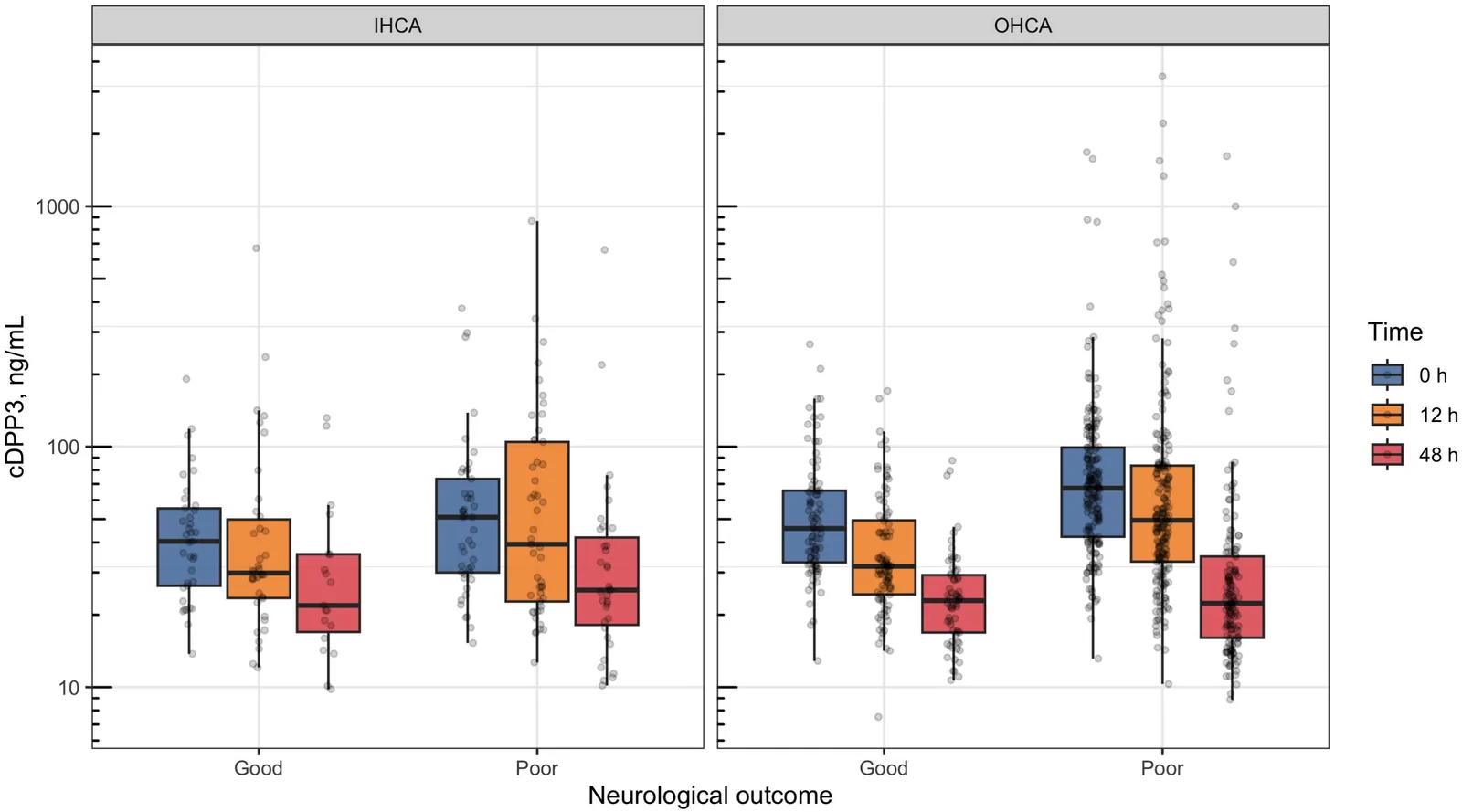
**Plasma cDPP3 by neurological outcome, stratified by arrest location. Boxplots show plasma cDPP3 concentrations at ICU admission (0 h), 12 h and 48 h for patients with good and poor outcome. Benjamini–Hochberg-adjusted *p*-values from Welch *t*-tests on log_10_ values: IHCA 0.28/0.28/0.67, OHCA <0.001/*<*0.001/0.15 at 0, 12 and 48 h, respectively**. *cDPP3, circulating dipeptidyl peptidase 3; CPC, Cerebral Performance Category; IHCA, in-hospital cardiac arrest; OHCA, out-of-hospital cardiac arrest*.

**Fig. 3:**
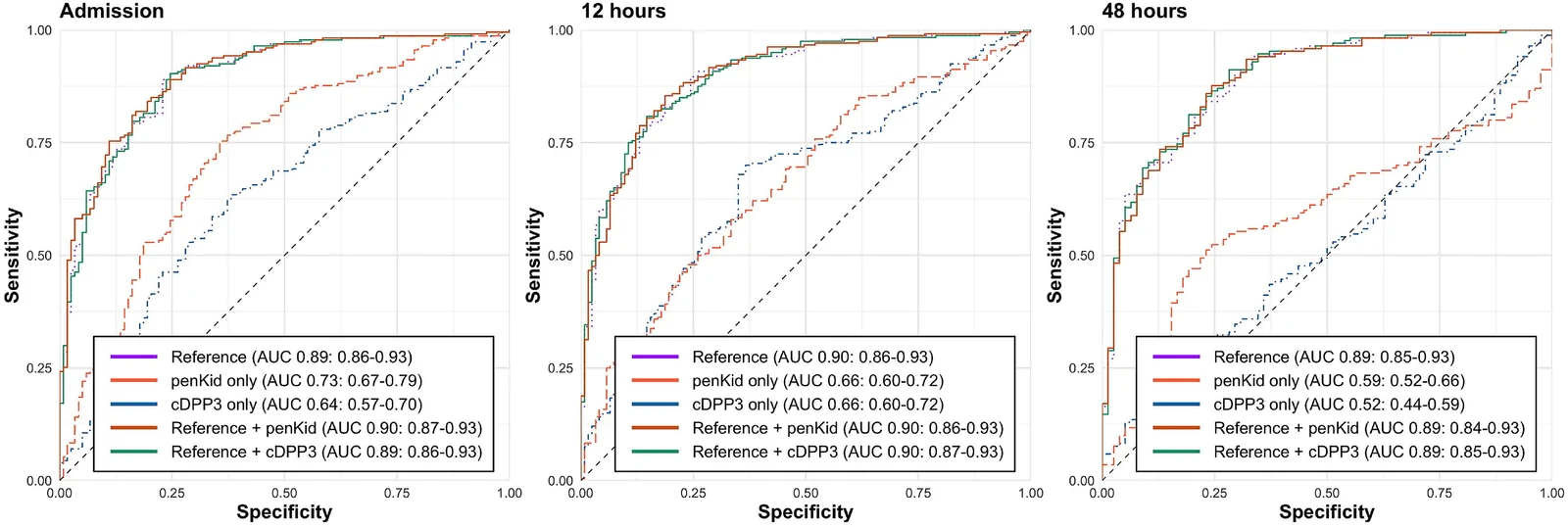
**Receiver operating characteristic curves (ROC) for poor neurological outcome for penKid and cDPP3 and the clinical reference model. The area under the receiver operating characteristic curve (AUC), with 95% confidence intervals, is shown for penKid and cDPP3 alone and in combination with clinical parameters (reference) on admission, at 12 h, and at 48 h**. *penKid, plasma proenkephalin A 119*–*159; cDPP3, circulating dipeptidyl peptidase 3; ref, reference*.

### Secondary outcome: 180-day mortality

Within 180 days of cardiac arrest, 266 (63.8%) had died with median survival of 4 days. Kaplan-Meier survival analyses stratified by biomarker tertiles are shown in Figure S4. Higher plasma penKid concentrations were associated with a lower 180-day survival (log-rank *p <* 0*.*001, at all time points). Similarly, higher cDPP3 concentrations were associated with lower survival on admission and 12 h (log-rank *p <* 0*.*001), but not at 48 h (log-rank *p* = 0*.*6). In Cox regression analyses, increasing penKid concentrations were associated with higher 180-day mortality at all time points, with the strongest associations observed at admission and 12 h. After multivariate adjustment, penKid remained independently associated with mortality on admission (adjusted HR 1.36, 95% CI 1.20–1.54, *p <* 0*.*001) and at 12 h (adjusted HR 1.33, 95% CI 1.17–1.51, *p <* 0*.*001), but not at 48 h. Elevated concentrations of cDPP3 were associated with mortality at admission and at 12 h in univariable analysis; however, after adjustment, only the 12-h measurement remained independently associated with mortality (adjusted HR 1.26, 95% CI 1.11–1.43, *p <* 0*.*001), see Table 3.

### Landmark analysis

Landmark analyses at 12 and 48 h yielded results consistent with the primary Cox regression analyses. PenKid remained associated with mortality at admission and 12 h, whereas cDPP3 remained associated with mortality at 12 h only. Detailed results are shown in Supplementary Tables S6 and S7.

### Exploratory cause-specific mortality analyses

Exploratory cause-specific mortality analyses are presented in Supplementary Table S10 and Figure S5, which showed similar associations of elevated biomarker concentrations with both neurological and non-neurological death.

### Sensitivity analysis of patients without WLST

In a prespecified sensitivity analysis excluding patients undergoing WLST, the direction and magnitude of associations were generally consistent with the primary analyses. Although some associations were numerically stronger, biomarker addition did not materially improve discrimination beyond the clinical reference model. Detailed results are shown in Supplementary Tables S4 and S5, as well as Supplementary Figure S3.

### Internal validation and model performance

Internal bootstrap validation demonstrated only minor optimism in the apparent AUCs, with optimism-corrected AUCs remaining high across all time points (Supplementary Table S8). Likelihood-ratio testing demonstrated incremental prognostic value of penKid beyond the reference model at admission and 12-h, but not at 48-h, whereas cDPP3 did not significantly improve model performance (Supplementary Table S9). The calibration plots showed good agreement between predicted and observed risks (Supplementary Figure S6).

## Discussion

In this multicentre serial analysis, early (0–12-h) plasma penKid was independently associated with poor neurological outcome and with 180-day mortality after adjustment for established clinical prognostic variables. cDPP3 showed independent association with 180-day mortality at 12-h in Cox regression. Neither biomarker significantly improved the prognostic abilities of the strong reference model for poor neurological outcome or for 180-day mortality. The trajectory analyses further support that the first 12 h after cardiac arrest are particularly important, as the differences in biomarker concentrations between outcome groups were greatest early and decreased thereafter, especially for penKid.

Several mechanisms may explain elevated plasma penKid post-cardiac arrest. It is a known marker of glomerular filtration and kidney dysfunction,^8^ which is common after cardiac arrest, making a renal contribution likely. Although creatinine was not included in the primary models because admission creatinine data were incomplete and adjustment could constitute over-adjustment given the close physiological relationship between penKid and renal function, sensitivity analyses including creatinine yielded similar findings (Supplementary Table S3). Since we did not include serial renal function measures in our analyses, the extent of renal involvement cannot be determined. Its levels are increased in other critical illnesses, suggesting that it may reflect overall illness severity rather than organ-specific injury.^11^, ^29^ A cerebral contribution is biologically plausible, as penKid concentrations are substantially higher in CSF than in plasma.^6^ Blood-brain barrier (BBB) disruption has been described within the first 24 h after cardiac arrest,^30^, ^31^ making early leakage into plasma possible. Further studies incorporating simultaneous plasma and CSF sampling are needed to clarify the origin of circulating penKid in this setting.

In many respects cDPP3 differs from penKid. Its association with outcome was strongest at 12 h after the cardiac arrest but was only independently associated with 180-day mortality in adjusted Cox-regression analysis, not with poor neurological outcome after adjusting for the clinical covariates. It is known to be released following cellular injury and has been linked to both circulatory and hemodynamic failure in the critically ill.^18^, ^19^, ^20^, ^21^ The results from our study might thus reflect ongoing post-resuscitation injury rather than neurological damage specifically, thus reflecting overall illness severity and progression after cardiac arrest rather than being a neuroprognostic biomarker.

Several exploratory analyses were performed to assess the robustness of the findings. Landmark analyses yielded results consistent with the primary analyses, suggesting that the weaker associations observed at 48 h were unlikely to be explained solely by survival bias. Sensitivity analyses excluding patients undergoing WLST and exploratory cause-specific mortality analyses also showed broadly similar patterns of association, although these findings should be interpreted cautiously because of potential selection bias and the challenges of attributing cause of death after cardiac arrest Detailed results are provided in the Supplementary Material.^32^

The reference model in our data showed high discriminatory performance (AUC of 0.89–0.90), which may explain why adding the biomarkers did not significantly improve discrimination in the combined model. This suggests that the prognostic information reflected by these biomarkers overlap with the established clinical variables. Their potential clinical role may thus be in a more specific settings not assessed in this study, like improving risk stratification in subgroups or complementing existing prognostic tools.

Internal validation demonstrated only minor optimism in the apparent AUCs, indicating limited overfitting of the clinical reference model. Although penKid provided statistically significant incremental prognostic information at admission and 12 h in likelihood-ratio analyses, this reflected improved model fit and did not translate into a meaningful improvement in AUC-based discrimination. Together with the calibration analyses, these findings support the robustness of the primary conclusion that the biomarkers were associated with outcome but added limited predictive value beyond established clinical predictors.

Neuronal biomarkers such as neurofilament light (NfL),^33^, ^34^, ^35^, ^36^ glial fibrillary acidic protein (GFAP),^24^ total tau (t-tau),^24^, ^37^ and S100B^2^ have demonstrated prognostic value after cardiac arrest. While NfL, GFAP, and t-tau reach peak prognostic performance at 24–48 h or later and reflect neuron- or glia-specific injury, S100B has shown good prognostic performance already at admission. Compared with these established biomarkers, the discriminatory performance of penKid and cDPP3 in the present study was modest.

In contrast to neuronal biomarkers, our data describe a different biomarker class: circulating peptides involved in systemic organ dysfunction that are present before and after cardiac arrest and whose post-arrest signal appears to increase within the first 12 h. Future studies measuring early peptide biomarkers alongside neuron-specific biomarkers in the same patients would be needed to determine whether multimarker approaches improve prognostic performance beyond individual biomarkers alone.^23^

We combined OHCA and IHCA in the primary regression analyses and adjusted for arrest location, while also presenting stratified descriptive and trajectory analyses; this approach is consistent with recent work suggesting that, after adjustment for relevant clinical covariates, outcomes differ less between these groups than raw comparisons suggest.^38^, ^39^ Biomarker signals were clearer in OHCA than in IHCA in stratified analyses; with the small IHCA subcohort (*n* = 99) the statistical power for the stratified analysis is limited.

### Strengths and limitations

Strengths of this study include a relatively large and heterogeneous multicenter cohort, with serial biomarker sampling, blinded batch analysis, and the inclusion of both neurological outcome and long-term mortality as endpoints. This improves generalisability and reliability of the findings. Missingness at 48 h was informative. Patients without a valid 48-h sample included both patients who died early and patients with short ICU stays compatible with early discharge. Thus, the 48-h analyses represent a selected subgroup of patients who remained available for sampling, and these findings should be interpreted cautiously. The sensitivity analysis was performed as well to address the potential influence of WLST on outcome associations.

There are several limitations in this study. It has a retrospective design, which carries a risk of inherent bias and a reliance on data quality and adequate documentation. Since patients with admission samples only were excluded, this may have introduced a selection bias by under-representing patients who died or were discharged from the ICU early. Missingness at 48 h was informative, as samples were often missing because of death or ICU discharge; analyses involving the 48-h biomarkers should therefore be interpreted cautiously. The high proportion of patients with WLST complicates the interpretation of neurological outcomes as these outcomes may to some extent reflect clinical decision-making in addition to biological injury. WLST practices vary between countries, centres, and over time, and our findings therefore reflect Swedish ICU practice during 2014–2018. Although participating centres followed contemporary guideline-based multimodal neuroprognostication strategies, WLST decisions were not protocolized and some variation between centres may have existed. Furthermore, neuroprognostication practices have evolved since the study period, including broader use of biomarkers and advanced neuroimaging,^1^ and WLST decisions in this cohort may therefore not fully reflect current clinical practice. Although a sensitivity analysis excluding patients with WLST was performed, this subgroup may not represent the entire post-cardiac arrest population, as some patients may have regained consciousness early or died from causes unrelated to hypoxic brain injury, making neurological prognostication unnecessary. Furthermore, neurological outcome assessments were performed between two and six months after cardiac arrest according to local follow-up practice rather than at a standardized time point. As neurological recovery may continue beyond the early post-arrest period, variability in the timing of outcome assessment may have resulted in some outcome misclassification. Although penKid is strongly related to kidney function, serial measures of renal dysfunction were not incorporated into the analysis, which limits assessment of the renal contribution to the plasma penKid concentrations. Finally, samples were stored frozen for variable periods of up to several years before analysis; while both biomarkers have shown good analytical stability under frozen storage and standardized, uniform sample handling makes outcome-related (differential) degradation unlikely, stability data covering the entire storage period are not available.

## Conclusion

In this study, early (≤12 h) penKid concentrations were independently associated with both poor neurological outcome and 180-day mortality after adjustment for established clinical prognostic variables, whereas 12-h cDPP3 concentrations were independently associated with mortality. However, neither biomarker meaningfully improved discrimination beyond a strong clinical reference model. These findings suggest that penKid and cDPP3 may provide complementary information regarding post-cardiac arrest pathophysiology, although their incremental prognostic value appears limited and should be confirmed in future studies using standardized outcome assessment.

## CRediT authorship contribution statement

**Bergþóra Þorgeirsdóttir:** Writing – original draft, Visualization, Software, Investigation, Formal analysis, Data curation. **Anna Lybeck:** Writing – review & editing, Supervision, Data curation. **Hans Friberg:** Writing – review & editing, Supervision, Resources, Conceptualization. **Attila Frigyesi:** Writing – review & editing, Writing – original draft, Validation, Supervision, Software, Resources, Methodology, Funding acquisition, Formal analysis, Conceptualization.

## Ethics approval and consent to participate

This study was approved by the Regional Ethical Review Board in Lund, Sweden (registration numbers 2014–47 and 2022–02681–01). Patients and their next-of-kin were given the option to opt out of inclusion, and written informed consent was obtained from patients who regained cognitive ability to consent during follow-up.

## Consent for publication

Not applicable.

## Declaration of generative AI and AI-assisted technologies in the writing process

During preparation of this manuscript, the authors used ChatGPT (OpenAI) for language editing and formatting support. The authors reviewed and edited the content and take full responsibility for the content of the manuscript.

## Funding

**AF:** Regional research support, Region Skåne #2022-1284; Governmental funding of clinical research within the Swedish National Health Service (ALF) #2022:YF0009 and #2022-0075; Crafoord Foundation grant number #2021-0833; Lions Skåne research grants; Skåne University Hospital grants; Swedish Heart and Lung Foundation (HLF) #20220352 and #2022-0458.

## Role of the funding source

The funders had no role in study design, data collection, data analysis, data interpretation, writing of the manuscript, or the decision to submit the article for publication.

## Data availability

The datasets generated and analysed during the current study are not publicly available due to limitations in the ethical approval of the study and data management policies of Region Skåne. However, they are available from the corresponding author on reasonable request.

## Declaration of competing interest

The authors declare the following financial interests/personal relationships which may be considered as potential competing interests: Attila Frigyesi reports financial support was provided by Region Skåne. Attila Frigyesi reports financial support was provided by Swedish National Health Service. Attila Frigyesi reports financial support was provided by Crafoord Foundation. Attila Frigyesi reports was provided by Lions Skåne. Attila Frigyesi reports financial support was provided by Swedish Heart and Lung Association. If there are other authors, they declare that they have no known competing financial interests or personal relationships that could have appeared to influence the work reported in this paper.

## Appendix A Supplementary material

Supplementary Data 1 Additional tables and figures presenting patient characteristics, biomarker analyses, sensitivity analyses, survival analyses, and model validation.
