# Supplementary material for: Serial plasma proenkephalin A 119–159 and dipeptidyl peptidase 3 after cardiac arrest: association with long-term neurological outcome
Source: Resusc Plus. 2026 Sep 17;32:101496. doi: 10.1016/j.resplu.2026.101496 (PMC13639150; doi:10.1016/j.resplu.2026.101496)
Supplement: Supplementary file 1 [file mmc1.docx]

Supplementary material

Serial plasma proenkephalin A 119–159 and dipeptidyl peptidase 3 after cardiac arrest: association with long-term neurological outcome

**Authors**

Bergþóra Þorgeirsdóttir[^1^](https://orcid.org/0009-0006-4514-6937)^,2*^, Anna Lybeck [^1^](https://orcid.org/0000-0001-7574-3958)^,3^, Hans Friberg [^1^](https://orcid.org/0000-0002-5588-0098)^,2^ and Attila Frigyesi [^1^](https://orcid.org/0000-0002-0155-4828)^,3^

**Affiliations**

^1^ Department of Clinical Sciences, Anaesthesiology and Intensive Care, Lund University, SE-22185 Lund, Sweden.

^2^ Skåne University Hospital, Department of Intensive and Perioperative Care, SE-20502 Malmö, Sweden.

^3^ Skåne University Hospital, Department of Intensive and Perioperative Care, SE-22185 Lund, Sweden.

Correspondance: [bergthora.thorgeirsdottir@med.lu.se](mailto:bergthora.thorgeirsdottir@med.lu.se)

**Table of Contents**

**Supplementary Tables**

**Table S1.** Characteristics of the study population by arrest location .......................................3

**Table S2.** Univariable and multivariable logistic regression analyses for poor neurological outcome .................................................................................................................................. 5

**Table S3.** Sensitivity analysis evaluating the impact of creatinine adjustment on the discriminatory performance of penKid for poor neurological outcome ................................. 6

**Table S4.** Sensitivity analysis excluding patients undergoing WLST ........................................ 7

**Table S5.** ROC sensitivity analysis excluding patients undergoing WLST ................................ 8

**Table S6.** Landmark Cox regression analyses for 180-day mortality ....................................... 9

**Table S7.** Additive prognostic value of biomarkers in landmark Cox analyses ....................... 9

**Table S8.** Internal validation of logistic regression models ..................................................... 9

**Table S9.** Incremental prognostic value of biomarker-augmented logistic regression models ……………………………………………………………………………………………………………………………… 10

**Table S10:** Exploratory cause-specific Cox proportional hazards regression analyses for neurological and non-neurological death………………………………………………………………………….. 10

**Table S11:** Comparison of patients with and without a valid 48-hour penKid sample………..……….. 11

**Supplementary Figures**

**Figure S1.** Flowchart of the study population ....................................................................... 12

**Figure S2.** Biomarker trajectories according to arrest location and neurological outcome ................................................................................................................................ 12

**Figure S3.** ROC curves for prediction of poor neurological outcome after exclusion of WLST patients .................................................................................................................................. 13

**Figure S4.** Kaplan–Meier survival curves by biomarker tertile .............................................. 13

**Figure S5.** Cumulative incidence functions for neurological versus non-neurological death by biomarker level at admission ................................................................................................ 14

**Figure S6.** Calibration of the clinical reference model and biomarker-augmented models...14

# Supplementary tables:

Overall	IHCA	OHCA	p-value

| Number of patients, n (%) | 417 (100) | 99 (23.7) | 318 (76.3) |  |
| --- | --- | --- | --- | --- |
| General characteristics  Female sex, n (%) | 115 (27.6) | 36 (36.4) | 79 (24.8) | 0.03 |
| Age, years, median (IQR) | 69 (59–76) | 71 (60–77) | 67 (59–76) | 0.08 |
| Cardiac arrest variables  Time to ROSC, min, median (IQR) | 20 (12–36) | 10 (6–20) | 25 (15–40) | *<*0.001 |
| Witnessed cardiac arrest, n (%) | 330 (79.1) | 82 (82.8) | 248 (78.0) | 0.33 |
| Bystander CPR, n (%) | 335 (80.3) | 99 (100) | 236 (74.2) | *<*0.001 |
| Adrenaline, n (%) | 336 (80.6) | 82 (82.8) | 254 (79.9) | 0.56 |
| Shockable rhythm, n (%) | 194 (46.7) | 21 (21.4) | 173 (54.6) | *<*0.001 |
| Admission motor GCS, median (IQR) | 1 (1–3) | 3 (1–5) | 1 (1–2) | *<*0.001 |
| Admission pupillary reflex absent, n (%) | 147 (36.6) | 19 (20.7) | 128 (41.3) | *<*0.001 |
| Biomarkers on admission penKid (pmol/L), median (IQR) | 123 (81–179) | 122 (74–174) | 123 (82–180) | 0.64 |
| cDPP3 (ng/mL), median (IQR) | 55 (36–83) | 44 (28–63) | 60 (39–89) | *<*0.001 |
| Lactate (mmol/L), median (IQR) | 8.1 (4.9–10.9) | 6.7 (4.7–9.9) | 8.6 (5.2–11.4) | 0.01 |
| pH, median (IQR) | 7.18 (7.01–7.27) 7.18 (7.01–7.28) 7.18 (6.99–7.27) | | | 0.39 |
| Biomarkers at 12 hours penKid (pmol/L), median (IQR) | 102 (71–168) | 102 (64–167) | 101 (72–167) | 0.39 |
| cDPP3 (ng/mL), median (IQR) | 42 (27–75) | 32 (23–81) | 44 (29–75) | 0.07 |
| Biomarkers at 48 hours penKid (pmol/L), median (IQR) | 62 (42–105) | 75 (46–110) | 61 (42–100) | 0.37 |
| cDPP3 (ng/mL), median (IQR) | 23 (16–34) | 25 (18–39) | 23 (16–31) | 0.26 |
| Previous medical history  Myocardial infarction, n (%) | 69 (16.5) | 19 (19.2) | 50 (15.7) | 0.44 |
| Congestive heart failure, n (%) | 76 (18.2) | 26 (26.3) | 50 (15.7) | 0.02 |
| Hypertension, n (%) | 168 (40.3) | 48 (48.5) | 120 (37.7) | 0.06 |
| Liver disease, n (%) | 10 (2.4) | 4 (4.0) | 6 (1.9) | 0.26 |
| Diabetes, insulin dependent, n (%) | 50 (12.0) | 19 (19.2) | 31 (9.7) | 0.02 |
| Diabetes, non-insulin, n (%) | 53 (12.7) | 19 (19.2) | 34 (10.7) | 0.04 |
| Cerebrovascular disease, n (%) | 40 (9.6) | 15 (15.2) | 25 (7.9) | 0.05 |
| Solid tumour, n (%) | 46 (11.0) | 17 (17.2) | 29 (9.1) | 0.04 |
| Renal disease, n (%) | 41 (9.8) | 17 (17.2) | 24 (7.5) | 0.01 |
| Dementia / cognitive impairment, n (%) | 19 (4.6) | 6 (6.1) | 13 (4.1) | 0.41 |
| Patient outcome  ICU LOS, days, median (IQR) | 3.2 (1.8–5.0) | 3.2 (1.4–4.9) | 3.2 (1.9–5.0) | 0.60 |
| Hospital LOS, days, median (IQR) | 7 (3–13) | 9 (4–20) | 6 (3–12) | *<*0.01 |
| WLST, n (%) | 209 (50.1) | 34 (34.3) | 175 (55.0) | *<*0.001 |
| 180-day mortality, n (%) | 266 (63.8) | 53 (53.5) | 213 (67.0) | *<*0.001 |

**Table S1:** Characteristics of the study population by arrest location. *Values are presented as median (IQR) for continuous variables and n (%) for categorical variables. Continuous variables were compared using the Mann-Whitney U test and categorical variables using Fisher’s exact test. IHCA, in-hospital cardiac arrest; OHCA, out-of-hospital cardiac arrest; ROSC, return of spontaneous circulation; CPR, cardiopulmonary resuscitation; GCS, Glasgow Coma Scale; ICU, intensive care unit; LOS, length of stay; WLST, withdrawal of life-sustaining therapies; CPC, Cerebral Performance Category.*

Univariable	Multivariable

Variable	OR	95% CI	p-value	OR	95% CI	p-value

| Admission log_10_ penKid, per 1 SD | 2.49 | 1.87–3.32 | *<*0.001 | 1.62 | 1.15–2.28 | 0.01 |
| --- | --- | --- | --- | --- | --- | --- |
| log_10_ cDPP3, per 1 SD | 1.68 | 1.29–2.19 | *<*0.001 | 1.12 | 0.76–1.66 | 0.57 |
| Age (per 10 years) | 1.23 | 1.05–1.44 | 0.01 | 1.67 | 1.28–2.17 | *<*0.001 |
| Male sex | 0.71 | 0.43–1.19 | 0.19 | 0.99 | 0.48–2.05 | 0.98 |
| Time to ROSC (per 10 min) | 1.67 | 1.40–1.99 | *<*0.001 | 1.62 | 1.28–2.05 | *<*0.001 |
| Motor GCS | 0.56 | 0.48–0.65 | *<*0.001 | 0.59 | 0.48–0.72 | *<*0.001 |
| Shockable rhythm | 0.19 | 0.12–0.32 | *<*0.001 | 0.09 | 0.04–0.21 | *<*0.001 |
| Pupillary reflex present | 0.21 | 0.12–0.36 | *<*0.001 | 0.62 | 0.30–1.31 | 0.21 |
| Bystander CPR | 0.46 | 0.24–0.85 | 0.01 | 0.86 | 0.37–1.99 | 0.72 |
| IHCA | 0.52 | 0.31–0.88 | 0.01 | 0.51 | 0.21–1.24 | 0.14 |
| 12 hours log_10_ penKid, per 1 SD | 1.78 | 1.38–2.28 | *<*0.001 | 1.50 | 1.08–2.09 | 0.02 |
| log_10_ cDPP3, per 1 SD | 2.00 | 1.49–2.68 | *<*0.001 | 1.35 | 0.93–1.95 | 0.11 |
| Age (per 10 years) | 1.19 | 1.02–1.38 | 0.03 | 1.72 | 1.34–2.19 | *<*0.001 |
| Male sex | 0.64 | 0.39–1.05 | 0.08 | 0.82 | 0.41–1.66 | 0.59 |
| Time to ROSC (per 10 min) | 1.69 | 1.42–2.01 | *<*0.001 | 1.71 | 1.36–2.15 | *<*0.001 |
| Motor GCS | 0.55 | 0.47–0.64 | *<*0.001 | 0.57 | 0.47–0.70 | *<*0.001 |
| Shockable rhythm | 0.20 | 0.12–0.31 | *<*0.001 | 0.09 | 0.04–0.19 | *<*0.001 |
| Pupillary reflex present | 0.19 | 0.11–0.33 | *<*0.001 | 0.56 | 0.27–1.14 | 0.11 |
| Bystander CPR | 0.32 | 0.16–0.62 | *<*0.001 | 0.63 | 0.26–1.51 | 0.30 |
| IHCA | 0.63 | 0.38–1.05 | 0.08 | 0.75 | 0.32–1.75 | 0.51 |
| 48 hours log_10_ penKid, per 1 SD | 1.38 | 1.03–1.84 | 0.03 | 1.19 | 0.77–1.84 | 0.44 |
| log_10_ cDPP3, per 1 SD | 1.23 | 0.91–1.66 | 0.18 | 1.28 | 0.87–1.90 | 0.22 |
| Age (per 10 years) | 1.19 | 0.97–1.45 | 0.10 | 1.81 | 1.30–2.50 | *<*0.001 |
| Male sex | 0.69 | 0.37–1.29 | 0.25 | 0.85 | 0.36–2.03 | 0.71 |
| Time to ROSC (per 10 min) | 1.53 | 1.24–1.90 | *<*0.001 | 1.59 | 1.20–2.12 | *<*0.001 |
| Motor GCS | 0.51 | 0.42–0.62 | *<*0.001 | 0.47 | 0.36–0.62 | *<*0.001 |
| Shockable rhythm | 0.18 | 0.10–0.33 | *<*0.001 | 0.07 | 0.03–0.20 | *<*0.001 |
| Pupillary reflex present | 0.22 | 0.11–0.43 | *<*0.001 | 0.59 | 0.25–1.39 | 0.23 |
| Bystander CPR | 0.54 | 0.27–1.09 | 0.08 | 1.12 | 0.42–2.94 | 0.83 |
| IHCA | 0.74 | 0.39–1.43 | 0.38 | 0.56 | 0.19–1.64 | 0.29 |

**Table S2:** Univariable and multivariable logistic regression analyses for poor neurological outcome. Associations between plasma biomarker concentrations and poor neurological outcome (CPC 3-5) were evaluated at admission, 12 hours, and 48 hours after cardiac arrest. Multivariable models were adjusted for age, sex, bystander CPR, time to ROSC, motor GCS, shockable rhythm, pupillary reflex, and arrest location (IHCA versus OHCA). Biomarkers were log_10_-transformed and standardized; odds ratios (ORs) represent the effect per one standard deviation increase in biomarker concentration.

*CI, confidence interval; CPC, Cerebral Performance Category; CPR, cardiopulmonary resuscitation; cDPP3, circulating dipeptidyl peptidase 3; GCS, Glasgow Coma Scale; IHCA, in-hospital cardiac arrest; OHCA, out-of-hospital cardiac arrest; OR, odds ratio; penKid, plasma proenkephalin A 119–159; ROSC, return of spontaneous circulation; SD, standard deviation.*

| Time point Model n | | | Events AUC (95% CI) | | | *p* vs reference |
| --- | --- | --- | --- | --- | --- | --- |
| Admission | Reference  Reference + penKid  Reference + creatinine | 345  345  255 | | 227  227  169 | 0.89 (0.86–0.93)  0.90 (0.87–0.93)  0.92 (0.88–0.95) | – 0.16  0.27 |
|  | Reference + creatinine + penKid 255 | | | 169 | 0.92 (0.88–0.95) | 0.28 |
|  | penKid only | 345 | | 227 | 0.73 (0.67–0.78) | – |
| 12 h | Reference  Reference + penKid  Reference + creatinine | 363  363  258 | | 240  240  172 | 0.90 (0.86–0.93)  0.90 (0.86–0.93)  0.92 (0.88–0.95) | – 0.44  0.05 |
|  | Reference + creatinine + penKid 258 | | | 172 | 0.92 (0.88–0.95) | 0.79 |
|  | penKid only | 363 | | 240 | 0.66 (0.60–0.72) | – |
| 48 h | Reference  Reference + penKid  Reference + creatinine | 248  248  177 | | 170  170  128 | 0.89 (0.85–0.93)  0.89 (0.85–0.93)  0.93 (0.89–0.96) | – 0.77  0.21 |
|  | Reference + creatinine + penKid 177 | | | 128 | 0.93 (0.89–0.96) | 0.94 |
|  | penKid only 248 | | | 170 | 0.59 (0.52–0.66) | – |

**Table S3:** Sensitivity analysis evaluating the impact of creatinine adjustment on the discriminatory performance of penKid for poor neurological outcome. ROC curve analyses compared the clinical reference model, the reference model plus penKid, the reference model plus creatinine, and the reference model plus creatinine and penKid. Biomarker-augmented models were compared with the corresponding reference model using DeLong’s test. Values are presented as AUC with 95% confidence intervals (CI). *AUC, area under the receiver operating characteristic curve; CI, confidence interval; penKid, plasma proenkephalin A 119–159; ROC, receiver operating characteristic.*

| Time point Biomarker |  | Univariable logistic | | Multivariable logistic Multivariable Cox regression | |
| --- | --- | --- | --- | --- | --- |
|  |  | OR (95% CI) | p | OR (95% CI) p HR (95% CI) | p |
| Admission | penKid | 2.09 (1.42–3.07) | *<* 0*.*001 | 1.59 (0.99–2.56) 0.06 1.40 (1.09–1.80) | 0.01 |
| Admission | cDPP3 | 1.71 (1.21–2.42) | *<* 0*.*01 | 1.39 (0.82–2.35) 0.22 1.14 (0.83–1.57) | 0.42 |
| 12 h | penKid | 1.55 (1.10–2.17) | 0.01 | 1.33 (0.88–2.03) 0.18 1.28 (0.99–1.66) | 0.06 |
| 12 h | cDPP3 | 2.01 (1.39–2.91) | *<* 0*.*001 | 1.82 (1.12–2.94) 0.02 1.41 (1.08–1.84) | 0.01 |
| 48 h | penKid | 2.01 (1.25–3.23) | *<* 0*.*01 | 1.62 (0.86–3.06) 0.14 1.44 (0.94–2.22) | 0.09 |
| 48 h | cDPP3 | 1.58 (1.03–2.43) | 0.04 | 1.89 (0.97–3.67) 0.01 1.20 (0.87–1.65) | 0.27 |

**Table S4:** Sensitivity analysis excluding patients undergoing WLST. Multivariable logistic regression and Cox proportional hazards models were used to assess associations between biomarker concentrations and poor neurological outcome (CPC 3-5) and 180-day mortality, respectively. All models were adjusted for the same clinical covariates as in the primary analysis. HRs and ORs are reported per 1 SD increase in log_10_transformed biomarker concentration. *CI, confidence interval; CPC, Cerebral Performance Category; cDPP3, circulating dipeptidyl peptidase 3; HR, hazard ratio; OR, odds ratio; penKid, plasma proenkephalin A 119–159; WLST, withdrawal of life-sustaining therapies.*

| Time point | Model | n | Events | AUC (95% CI) | *p* vs reference |
| --- | --- | --- | --- | --- | --- |
| Admission | Reference | 169 | 51 | 0.85 (0.77–0.91) | – |
|  | Reference + penKid | 169 | 51 | 0.85 (0.78–0.91) | 0.85 |
|  | Reference + cDPP3 | 169 | 51 | 0.85 (0.78–0.92) | 0.51 |
|  | penKid only | 169 | 51 | 0.69 (0.60–0.78) | – |
|  | cDPP3 only | 169 | 51 | 0.63 (0.53–0.73) | – |
| 12 h | Reference | 176 | 53 | 0.85 (0.77–0.91) | – |
|  | Reference + penKid | 176 | 53 | 0.85 (0.77–0.91) | 0.86 |
|  | Reference + cDPP3 | 176 | 53 | 0.86 (0.78–0.92) | 0.44 |
|  | penKid only | 176 | 53 | 0.63 (0.54–0.72) | – |
|  | cDPP3 only | 176 | 53 | 0.66 (0.57–0.75) | – |
| 48 h | Reference | 106 | 28 | 0.85 (0.76–0.93) | – |
|  | Reference + penKid | 106 | 28 | 0.86 (0.77–0.93) | 0.51 |
|  | Reference + cDPP3 | 106 | 28 | 0.87 (0.78–0.94) | 0.46 |
|  | penKid only | 106 | 28 | 0.69 (0.57–0.81) | – |
|  | cDPP3 only | 106 | 28 | 0.56 (0.44–0.6) | – |

**Table S5:** ROC sensitivity analysis excluding patients undergoing WLST. Discriminatory performance for poor neurological outcome (CPC 3-5) was assessed using ROC curve analysis. Biomarker-augmented models were compared with the clinical reference model using DeLong’s test. Values are presented as AUC with 95% CI.*AUC, area under the receiver operating characteristic curve; CI, confidence interval; CPC, Cerebral Performance Category; ROC, receiver operating characteristic.*

| Time point | Biomarker | N | Deaths | HR (95% CI) | P-value |
| --- | --- | --- | --- | --- | --- |
| 0 h | penKid | 344 | 224 | 1.36 (1.20–1.54) | *<*0.001 |
| 0 h | cDPP3 | 344 | 224 | 1.12 (0.97–1.29) | 0.15 |
| 12 h | penKid | 362 | 238 | 1.33 (1.17–1.51) | *<*0.001 |
| 12 h | cDPP3 | 362 | 238 | 1.24 (1.09–1.42) | *<*0.001 |
| 48 h | penKid | 236 | 156 | 1.08 (0.90–1.29) | 0.40 |
| 48 h | cDPP3 | 236 | 156 | 0.94 (0.79–1.11) | 0.44 |

**Table S6:** Landmark Cox regression analyses for 180-day mortality. Associations between biomarker concentrations and 180-day mortality were evaluated using landmark Cox proportional hazards models at admission, 12 hours, and 48 hours. Hazard ratios are presented per 1-SD increase in log_10_-transformed biomarker concentration and adjusted for age, sex, time to ROSC, motor GCS score, shockable rhythm, pupillary reflexes, bystander CPR, and arrest location. Values are presented as HR with 95% CI.

*CI, confidence interval; GCS, Glasgow Coma Scale; HR, hazard ratio; ROSC, return of spontaneous circulatio; cDPP3, circulating dipeptidyl peptidase 3; penKid, plasma proenkephalin A 119–159.*

| Time point | Comparison | *χ*2 | P-value | BH-adjusted P |
| --- | --- | --- | --- | --- |
| 0 h | Reference vs Reference + penKid | 20.03 | *<*0.001 | *<*0.001 |
| 0 h | Reference vs Reference + cDPP3 | 2.21 | 0.14 | 0.21 |
| 12 h | Reference vs Reference + penKid | 17.52 | *<*0.001 | *<*0.001 |
| 12 h | Reference vs Reference + cDPP3 | 9.88 | *<*0.01 | *<*0.01 |
| 48 h | Reference vs Reference + penKid | 0.69 | 0.41 | 0.43 |
| 48 h | Reference vs Reference + cDPP3 | 0.62 | 0.43 | 0.43 |

**Table S7:** Additive prognostic value of biomarkers in landmark Cox analyses. The incremental prognostic value of penKid and cDPP3 beyond the clinical reference model was assessed using likelihood-ratio tests comparing nested landmark Cox proportional hazards models at admission, 12 hours, and 48 hours. Values are presented as likelihood-ratio test statistics (*χ*^2^) and corresponding P-values. *χ*^2^*, chi-square statistic; BH: Benjamini-Hochberg; cDPP3, circulating dipeptidyl peptidase 3; penKid, plasma proenkephalin A 119–159.*

| Time point | Model | N | Events | Apparent AUC | Optimism | Corrected AUC |
| --- | --- | --- | --- | --- | --- | --- |
| Admission | Reference | 345 | 227 | 0.895 | 0.010 | 0.884 |
| Admission | Reference + penKid | 345 | 227 | 0.901 | 0.012 | 0.889 |
| Admission | Reference + cDPP3 | 345 | 227 | 0.895 | 0.012 | 0.883 |
| 12 h | Reference | 363 | 240 | 0.897 | 0.012 | 0.885 |
| 12 h | Reference + penKid | 363 | 240 | 0.900 | 0.013 | 0.887 |
| 12 h | Reference + cDPP3 | 363 | 240 | 0.900 | 0.013 | 0.886 |
| 48 h | Reference | 248 | 170 | 0.892 | 0.019 | 0.873 |
| 48 h | Reference + penKid | 248 | 170 | 0.891 | 0.021 | 0.870 |
| 48 h | Reference + cDPP3 | 248 | 170 | 0.894 | 0.020 | 0.874 |

**Table S8:** Internal validation of logistic regression models. Apparent and optimism-corrected AUCs were estimated using bootstrap resampling with 500 repetitions. *AUC, area under the receiver operating characteristic curve; cDPP3, circulating dipeptidyl peptidase 3; penKid, plasma proenkephalin A 119–159.*

| Time point | Comparison | *χ*2 | p-value | BH-adjusted p |
| --- | --- | --- | --- | --- |
| Admission | Reference vs Reference + penKid | 8.05 | 0.005 | 0.027 |
| Admission | Reference vs Reference + cDPP3 | 0.34 | 0.560 | 0.560 |
| 12 h | Reference vs Reference + penKid | 5.78 | 0.016 | 0.049 |
| 12 h | Reference vs Reference + cDPP3 | 2.91 | 0.088 | 0.176 |
| 48 h | Reference vs Reference + penKid | 0.59 | 0.442 | 0.531 |
| 48 h | Reference vs Reference + cDPP3 | 1.58 | 0.208 | 0.313 |

**Table S9:** Incremental prognostic value of biomarker-augmented logistic regression models. The incremental prognostic value of penKid and cDPP3 beyond the clinical reference model was assessed using likelihood-ratio tests comparing nested logistic regression models. *BH, Benjamini–Hochberg; cDPP3, circulating dipeptidyl peptidase 3; LRT, likelihood-ratio test; penKid, plasma proenkephalin A 119–159.*

| Time point | Biomarker | N | Events | HR | 95% CI | p-value |
| --- | --- | --- | --- | --- | --- | --- |
| 0 h | penKid, neurological death | 370 | 167 | 1.54 | 1.36–1.75 | *<* 0*.*001 |
| 0 h | penKid, non-neurological death | 370 | 73 | 1.64 | 1.43–1.87 | *<* 0*.*001 |
| 0 h | cDPP3, neurological death | 370 | 167 | 1.38 | 1.19–1.60 | *<* 0*.*001 |
| 0 h | cDPP3, non-neurological death | 370 | 73 | 1.49 | 1.27–1.74 | *<* 0*.*001 |
| 12 h | penKid, neurological death | 386 | 175 | 1.47 | 1.29–1.68 | *<* 0*.*001 |
| 12 h | penKid, non-neurological death | 386 | 74 | 1.60 | 1.38–1.84 | *<* 0*.*001 |
| 12 h | cDPP3, neurological death | 386 | 175 | 1.37 | 1.20–1.57 | *<* 0*.*001 |
| 12 h | cDPP3, non-neurological death | 386 | 74 | 1.61 | 1.40–1.84 | *<* 0*.*001 |

**Table S10:** Exploratory cause-specific Cox proportional hazards regression analyses for neurological and non-neurological death. Associations between plasma biomarker concentrations and neurological and non-neurological death within 180 days after cardiac arrest were evaluated using cause-specific Cox proportional hazards models at admission and 12 hours after cardiac arrest. Neurological death was defined as death following withdrawal of life-sustaining therapy for poor neurological prognosis or brain death. Non-neurological death included deaths attributed to organ failure, comorbidity, ethical treatment limitations, or deaths occurring after hospital discharge. Biomarkers were log_10_-transformed; HRs are reported per 1 SD increase in log_10_-transformed biomarker concentration. These analyses were exploratory and should be interpreted cautiously given the challenges of assigning cause of death after cardiac arrest. *CI, confidence interval; cDPP3, circulating dipeptidyl peptidase 3; HR, hazard ratio; penKid, plasma proenkephalin A 119–159.*

| **Characteristic** | **No valid 48-h sample (n=154)** | **Valid 48-h sample (n=263)** | **p value** |
| --- | --- | --- | --- |
| Age, years | 69.7 (58.0–77.4) | 67.4 (59.7–75.4) | 0.441 |
| Female sex | 47 (30.5%) | 68 (25.9%) | 0.309 |
| OHCA | 109 (70.8%) | 209 (79.5%) | 0.056 |
| Shockable rhythm | 62 (40.5%) | 132 (50.4%) | 0.054 |
| Time to ROSC, min | 20.0 (10.0–40.0) | 20.0 (14.0–35.0) | 0.658 |
| Admission motor GCS | 1.0 (1.0–4.0) | 1.0 (1.0–3.0) | 0.047 |
| Pupillary reflex present | 97 (65.5%) | 158 (62.2%) | 0.521 |
| Bystander CPR | 127 (82.5%) | 208 (79.1%) | 0.445 |
| Poor neurological outcome (CPC 3–5) | 92 (59.7%) | 179 (68.1%) | 0.090 |
| 180-day mortality | 91 (59.1%) | 176 (66.9%) | 0.114 |
| Death within 48 h | 54 (35.1%) | 12 (4.6%) | <0.001 |
| ICU discharge within 48 h | 53 (34.4%) | 28 (10.6%) | <0.001 |
| ICU length of stay, days | 1.6 (1.0–2.8) | 4.0 (2.8–5.7) | <0.001 |

**Table S11:** Comparison of patients with and without a valid 48-hour penKid sample. Values are presented as median (IQR) or n (%), unless otherwise indicated. Continuous and ordinal variables were compared using the Wilcoxon rank-sum test and categorical variables using Fisher’s exact test. Admission motor GCS was analysed as an ordinal variable. Missing values were excluded from variable-specific comparisons.

*Abbreviations: CPC, Cerebral Performance Category; CPR, cardiopulmonary resuscitation; GCS, Glasgow Coma Scale; ICU, intensive care unit; IQR, interquartile range; OHCA, out-of-hospital cardiac arrest; ROSC, return of spontaneous circulation.*

# Supplementary figures:

All adults admitted to ICU

after cardiac arrest (n = 797)

Excluded (n = 373):

Missed inclusion: 117

No consent: 52

Missing data: 11

No valid biobank sample: 193

Patients with

1

valid biomarker

sample in SWECRIT biobank (n = 424)

Excluded (n = 7):

Admission sample only

(

no 12 h or 48 h sample

)

Analytic cohort (n = 417)

1

serial sample at 12 h or 48 h

Out-of-hospital cardiac arrest

(

OHCA, n

= 318)

In-hospital cardiac arrest

(

IHCA, n

= 99)

**Figure S1**: Flowchart of the study population. *OHCA, out-of-hospital cardiac arrest; IHCA, in-hospital cardiac arrest.*

**Figure S2:** Biomarker trajectories according to arrest location and neurological outcome. Thin lines represent individual patients and bold curves represent model-fitted trajectories with 95% confidence bands. Biomarker concentrations are shown on a log_10_ scale across admission, 12 hours, and 48 hours after cardiac arrest. Time × outcome interactions from mixed-effects models showed significant differences in OHCA patients (penKid *p* =0*.*02; cDPP3 *p* =0*.*03), but not in IHCA patients (penKid *p* =0*.*73; cDPP3 *p* =0*.*94). *penKid, proenkephalin A 119-159; cDPP3, circulating dipeptidyl peptidase 3; CA, cardiac arrest; CPC, Cerebral Performance Category.*

**Figure S3**: ROC curves for prediction of poor neurological outcome after exclusion of WLST patients. Discriminatory performance of penKid, cDPP3, and the clinical reference model at admission, 12 hours, and 48 hours after cardiac arrest. *WLST, withdrawal of life-sustaining therapies; penKid, plasma proenkephalin A 119-159; cDPP3, circulating dipeptidyl peptidase 3; ref, reference.*

**Figure S4:** Kaplan–Meier survival curves by biomarker tertile. Survival probabilities during the 180 days after cardiac arrest for patients stratified into tertiles of plasma penKid and cDPP3 at admission, at 12 hours and 48 hours after the arrest. Shaded bands show 95% confidence intervals. *p*-values are from the log-rank test across tertiles. *penKid, proenkephalin A 119–159; cDPP3, circulating dipeptidyl peptidase 3.*

**Figure S5:** Cumulative incidence functions for neurological versus nonneurological death, by biomarker level at admission. Patients are stratified by the median biomarker value at admission. Solid lines: cumulative incidence of neurological death (defined as death after withdrawal of life-sustaining therapies for poor neurological prognosis or after declaration of brain death). Dashed lines: cumulative incidence of non-neurological death (all other deaths). *penKid, proenkephalin A 119–159; cDPP3, circulating dipeptidyl peptidase 3.*

**Figure S6:** Calibration of the clinical reference model and biomarkeraugmented models. Calibration plots compare observed and predicted probabilities of poor neurological outcome (CPC 3-5) across deciles of predicted risk at admission, 12 hours, and 48 hours after cardiac arrest. The dashed diagonal line represents perfect agreement between predicted and observed risk. *CPC, Cerebral Performance Category; cDPP3, circulating dipeptidyl peptidase 3; penKid, plasma proenkephalin A 119–159.*
